# Supplementary material for: In Vitro Antimicrobial Effects and Inactivation Mechanisms of 5,8-Dihydroxy-1,4-Napthoquinone
Source: Antibiotics (Basel). 2022 Nov 3;11(11):1537. doi: 10.3390/antibiotics11111537 (PMC9687054; doi:10.3390/antibiotics11111537)
Supplement: Supplementary file 1 [file antibiotics-11-01537-s001.zip › antibiotics-1988160-supplementary.pdf]

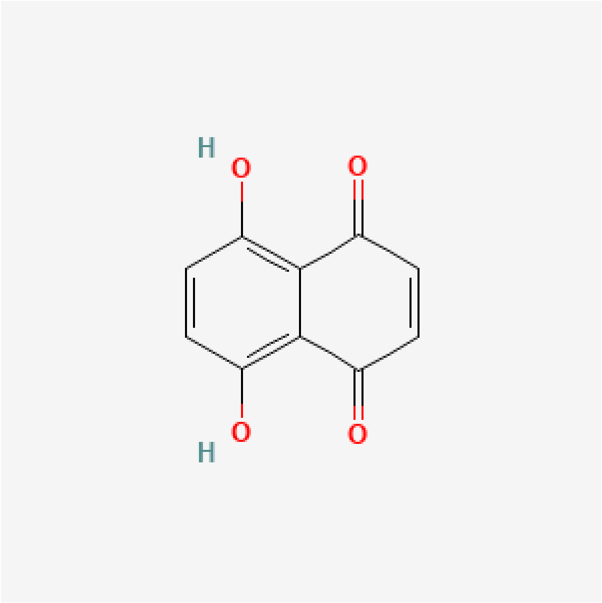

Figure S1. Structure of 5,8-dihydroxy-1,4-NQ (Naphthazarin).
(https://pubchem.ncbi.nlm.nih.gov/compound/5_8-Dihydroxy-1_4 naphthoquinone#section=Structures).
